# Can care of elderly be measured? A method for estimating the individual care of recipients in community health care

**DOI:** 10.1186/1471-2318-6-14

**Published:** 2006-09-19

**Authors:** Kajsa BE Thorsell, Berit M Nordström, Per Nyberg, Bengt V Sivberg

**Affiliations:** 1 Department of Health Sciences, Section of Nursing, Faculty of Medicine, Lund University, Box 157, Baravägen 3, SE 221 00 Lund, Sweden; 2 Municipality of Hassleholm, Section of elderly, Löjtnant Granlunds väg 14, SE- 28152 Hassleholm, Sweden

## Abstract

**Background:**

Almost every country in the Western world has great difficulties allocating enough financial resources to meet the needs in the care of the increasing elderly population. The main problem is common to all countries and concerns the efforts to meet elderly persons' needs on an individual level while still maintaining society's responsibility for distributing justice. The aim of this study is to elaborate an instrument for measuring the quality of individual care and staff's working time in order to allocate public resources fairly. The present study gives an account of a new classification system named TiC (Time in Care), indicating how it can be used most effectively and also investigating the validity and reliability of the system.

**Methods:**

All recipients in 13 sheltered homes for elderly care (n = 505) in a Swedish municipality were surveyed regarding the care they needed, in dimensions of General Care, Medical Care, Cognitive Dysfunction and Rehabilitation, and the time required. Construct validity was assessed by means of factor analysis. The inter-rater agreement of two raters concerning 79 recipients was measured using weighted Kappa. The stability of the instrument and its sensitivity to change were investigated through test-retest reliability measurements, conducted once a month during a six-month period. The content validity of the instrument was also assessed.

**Results:**

Factor analysis resulted in a reduction of the number of items from 25 to 16 in three dimensions: General Care, Medical Care and Cognitive Dysfunction. The Kappa analysis showed satisfactory to excellent inter-rater agreement. The care need scores were basically stable but showed sensitivity to change in health status.

**Conclusion:**

The instrument was found to be useful and reliable for assessing individual needs in community health care.

## Background

Almost every country in the Western world has great difficulties allocating enough financial resources to meet the needs in the care of the increasing elderly population. The main problem is common to all countries and concerns the efforts to meet elderly persons' needs on an individual level while still maintaining society's responsibility for distributing justice. The aim of this study is to elaborate an instrument for the measurement of the quality of individual care and staff's working time in order to allocate public resources fairly. Such an instrument needs to be easy to use, be clearly accepted, and be anchored in the staff of the health promoting-organization [1,2].

Since the 1980s, patient classification systems such as the Katz ADL index [3] and the Resident Assessment Instrument (RAI) [4] are uniform assessment systems used to assess and plan the care of residents in virtually all U.S. nursing homes. The RAI instrument became a federally mandated system as a part of a comprehensive set of nursing home reforms passed by the U.S. Congress in the Omnibus Budget Reconciliation Act of 1987 (OBRA '87) [4]. Athlin et al [5] and Kelleher [6] describe the validation of some instruments, and a model called Zebra has been employed by Levenstam [7,8] in order to measure the workload in hospitals. Fagerström [9] has recently described a factor-model evaluation system, used in Finland since 2000, involving the use of specific factors or indicators describing the patient's needs for care.

The instrument used in this study has been developed during the last four years in a municipality in southern Sweden. The original instrument enables a comprehensive assessment to be made of the total needs of each recipient, of how the work of the staff should best be allocated to the direct and indirect care of recipients and of other activities related to the unit.

## Methods

The present study aims to give an account of a new classification system named TiC (Time in Care), indicating how it can be used most effectively, also investigating the validity and reliability of the system. The design of the study was prospective and longitudinal, with an assistant and a registered nurse observing a defined cohort of care recipients.

The original TiC-O instrument (revised in the course of the study) used here consists of two parts. The first part, TiC-O for need (TiC-O_n_), measures the care needs of the individual recipient in terms of the dimensions *General Care, Medical Care, Cognitive Dysfunction*, and *Rehabilitative Activities*, a total 25 items (Table 1).

**Table 1:** Items of the four dimensions in TiC-O_n_. The recipients' needs in the four dimensions.

Common Care	Medical Care	Cognitive Dysfunction	Rehabilitation
Nutrition	Injection	Temper	Prophylaxis of contractures
Washing upper body	Wound treatment	Mood	Movement and walking exercises
Washing lower body	Pressure sores	Alarm	Training by a physiotherapist
Toilet visits	Administration of drugs	Orientation	Social activity
Dressing/Undressing	Taking of specimens	Social communication	
Shower/Bath	Catheter or stoma	Verbal communication	
Mobilization	Special care		
Observation			

The total of 25 items included were rated for each recipient on a 1–4 scale as follows: (1) minimal need of care, (2) moderate need of care, (3) considerable and recurrent need of care, and (4) total or nearly total dependence upon care. The second part, TiC-O for time (TiC-O_t_), measures the time input required for care, divided into three areas: direct input of time, indirect input of time and time input related to the unit.

Data collection took place between January 2001 and December 2003. The sample comprised 560 care recipients in a municipality in southern Sweden living in 13 sheltered homes for elderly care. Each home was divided into units of about ten recipients each. Fifty-five care recipients in addition to the 560 were not available for rating due to organizational changes.

A manual [10] specifying the different levels of care a recipient could need in each item was prepared as a help to the nursing staff in assessing the care in the first part of the instrument (TiC-O_n_). The staff was trained by the first author to understand the criterion of each item in the manual before the assessment took place. Each care recipient had a particular staff member in charge of his/her care who made the assessment together with a registered nurse. Assessment of the care needs was conducted before observations were made of the time input needed for care of the recipient. Specially designed software was used to facilitate the recording of these assessments [11].

The TiC-O_t _was performed by the first author in each unit during two days, and each day was divided into a day shift 7.00–14.00 and an evening shift 14.00–21.00. Each shift was divided into a number of operations, and the time needed for each shift was summed up at the end of the day by the first author. Each shift was comprised of 40 observations/shift for each of the 505 recipients living in the 13 sheltered homes.

The study was performed for the purposes of quality assurance and the care recipients and their relatives were verbally informed about the study. They have all given informed consent to participation. After examination the Regional Ethics Committee, Faculty of Medicine at Lund University (LU-321-03), has approved the study and judged that no formal inquiry was needed, as the study could be considered a part of the daily work with quality assurance. Full consideration has been given to the Declaration of Helsinki, which states that full information must be conveyed to the recipients.

All statistical analyses were performed using SPSS 11.5 (SPSS Inc, Chicago, USA). Construct validity was assessed by means of factor analysis (principal component analysis with varimax rotation). The criterion to be met by items to be included in the factor structure was their having a factor loading of > 0.4 [12]. Spearman's Rho was calculated to assess the relationships between the four dimensions of the instrument. For each of the items remaining in the instrument after factor analysis an inter-rater comparison was performed on 78 recipients. Each care recipient was rated twice during two days by a nurse assistant in charge and by the first author. A weighted Kappa (Κ_w_) was calculated according to Altman [12]. The stability and sensitivity of TiC-O were assessed by means of test-retest measurements each month for a period of six months. The care recipients were also evaluated each month over a six-month period in order to examine the criterion-related validity of TiC-O_n_. Registered scores were compared to the recipients' medical records to evaluate the instrument's capacity to detect and reveal changes in caring needs. During the whole period the medical records were blinded to the rater. The content validity of TiC-O was evaluated in repeated discussions with experienced staff members.

## Results

### Factor analysis and construct validity

Factor analysis resulted in a reduction of the number of items from 25 to 16, indicating that the four dimensions could be reduced to three. All concerned the need of care: *General Care *(9 items: mean 2.55 range 1.75–3.43), *Medical Care *(4 items: mean 1.20, range 1.12–1.26) and *Cognitive Dysfunction *(3 items: mean 1.20, range 0.35–1.78). Of the original 25 items nine could be eliminated because of failure to meet the factor-loading criterion or because of their loading on more than one factor (Table 2).

**Table 2:** Factor analysis.

	Factors			
		
Variables	General Care	General Care	Cognitive Dysfunction	Communalities
Dressing/Undressing	.919			.828
Toilet visits	.907			.876
Mobilization	.878			.795
Washing lower body	.870			.800
Washing upper body	.798			.872
Shower/Bath	.741			.582
Observation	.727			.617
Nutrition	.683			.731
Social activities	.582			.434
Taking of specimens		.875		.499
Injections		.779		.521
Pressure sores		.532		.562
Catheter or stoma		.511		.506
Verbal communication			.677	.704
Orientation			.657	.497
Social communication			.628	.486
% variance explained	40.6	13.8	8.2	
Cronbach's alpha	0.87	0.41	0.63	= 0.85

Factor analysis (principal component) yielding three factors: General care, Medical Care and Cognitive Dysfunction.

Correlation between these three factors in terms of Spearman's Rho were all significant, 0.124 between Medical Care and Cognitive Dysfunction, 0.572 between General Care and Cognitive Dysfunction, and 0.294 between Medical Care and General Care. In the revised 16-item TiC instrument (TiC-R), the total scores on the first part (TiC-R_n_), concerned with care needs as a whole, ranged from 0 to 68 points. The range of scores for each of the four levels of care needs is shown in Table 3.

**Table 3:** Levels of care. Level of care needs in point/level and time/level.

Level	Characterization	Point/level	Time in minutes/level
1	Minimal need of care	0–20	0–30
2	Moderate need of care	21–33	31–90
3	Recurrent and demanding need of care	34–45	91–150
4	Total or near dependence upon care	46-	151-

The mean score total for care needs in the 13 sheltered living homes as a whole was 33.35 (sd 9.57), the means for the separate homes ranging from 26.8 to 42.5 points. Eleven percent of the care recipients were classified as having a minimal need of care (mean score 19 points), 39% as having a moderate need of care (mean score 27 points), 39% a recurrent and demanding need of care (mean score 39 points), and 11% as being totally or nearly dependent on receiving care (mean score 48 points).

### Inter-reliability and stability

The mean of the weighted Kappa (Κ_w_) for the dimensions (n = 78) was 0.85, showing close overall agreement between the two raters (cf. Altman, p. 404). The Κ_w _values ranged from 0.67 to 0.98, the lowest value (0.67) pertaining to the *Medical Care *item concerned with the need of prophylaxis for pressure sores, the highest value (0.98) pertaining to the *General Care *item concerned with the need of help generally.

In order to evaluate the stability of the ratings, repeated assessments with TiC were performed monthly during six months. The health status of ten care recipients was followed during the same period to ascertain stable health status and changed health status. During the first three months the assessment was stable and in accordance with absence of registrations of changed health status. During the final three months, three care recipients had decreased health status, as demonstrated by an assessed increased need of care and documented in the medical records (Table 4).

**Table 4:** Measured care points.

Month	1	2	3	4	5	6
Recipients	Points	Points	Points	Points	Points	Points
1	6	6	6	6	6	6
2	13	13	13	13	13	18
3	21	21	21	21	21	21
4	8	8	8	8	8	8
5	9	9	9	9	9	9
6	15	15	15	15	15	15
7	7	7	7	12	12	12
8	17	17	17	17	17	17
9	5	5	5	13	13	13
10	4	4	4	4	45	*

Measured points in needed care during six months in a unit (n = 10). Points are given for each recipients in each month.

* The recipient has gone "ad mortum"

**Figure 1:**
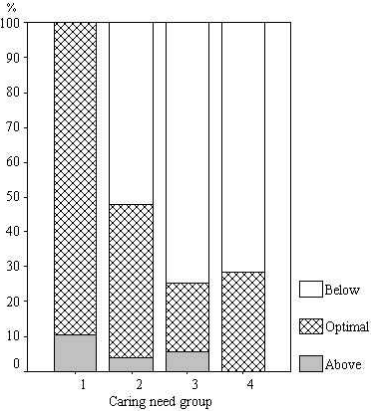
**Actual time input**. Actual time input of the nursing staff in direct care shown in relation to the percentages of recipients adjudged to be receiving optimal, below and above optimal care in terms of time input.

For the nursing homes, the overall percentages of time between 7 am and 9 pm/day the nursing staffs spent as a whole were found to be 61% for direct care, 34% for indirect care, and 5% for ward-related activities. The time spent on direct care was divided into four sets of time intervals, which can be correlated in a cross-tabulation between the four levels. Figure 1 shows the actual time input of the nursing staff in direct care shown in relation to the percentages of recipients.

At level-1, 10% of the recipients were considered to receive more than the optimal amount of time in the direct care given to them, whereas at level-2 52% were considered to receive a below-optimal amount of time, there being 74% in this category at level-3, and 71% at level-4.

## Discussion

Although there are well-established instruments of considerable complexity in this area such as the Katz ADL index and the Resident Assessment Instrument (RAI), most of them contain so many items that excessive time is needed to complete them, with the accompanying risk of some questions being avoided. It was seen as important to develop a satisfactory instrument that would take only a few minutes to complete.

A major question was how the four dimensions pertaining to the first part of the instrument were related to each other. In the factor analysis it was found that some items, which had a relatively low factor loading (less than 0.40), could be excluded from the original TiC-O_n_, making it simpler to use and to understand, a matter DeGroot [2] emphasized as being important. The four original dimensions could also be reduced to three (*General Care, Medical Care *and *Cognitive Care*). However when the four factors were reduced to three the value of Cronbach's alpha showed a very low value (0.41) in *Medical Care*. This dimension could probably also be reduced, but since *Medical Care *is a very important dimension to measure it was decided to include this dimension in the instrument.

In order to test the inter-rater reliability of the instrument, two persons performed independent assessments of each member of a group of care recipients. The Kappa measure employed showed close agreement between the two raters [13]. There were potential difficulties in testing the reliability and validity of TiC in a group of recipients, many of whom could be undergoing changes in health status. To circumvent these difficulties, an evaluation of the care needs of ten of the 78 recipients was performed monthly during a six-month period. Close agreement between successive evaluations was found for most of these persons. During the second half of the period there was a lack of agreement for some of them, who displayed an increase in need of care and a worsening of health status. These findings can be interpreted as showing both the stability of the instrument over time and its sensitivity to changes in health status.

The 13 sheltered homes were shown to differ in the average care needs of the recipients residing there. Despite these differences, the size of the staff was the same in each of the units. It can thus be concluded that the overall planning for the homes did not take adequate account of the needs of the recipients. The utilization and communication of relevant information concerning the care needs of individual recipients, together with planning in line with this, should clearly be handled more satisfactorily.

Regarding the question whether direct care in time of each recipient was related to the adjudged need of care for each recipient (Figure 1), it was found that the time input appeared optimal for those recipients in whom the largest amount of time was invested in care (level-1). Recipients in whom considerably less time was invested (level-3 and level-4) the percentage of recipients for whom the time input was adjudged suboptimal was particularly large. Further investigation, aimed at determining how an optimal level of care can best be provided, is called for.

## Conclusion

The reduced version of the test instrument, TiC-R, appears to be quite satisfactory for assessing the care needs of the elderly using staff competence and economic resources according to claims of societal justice.

## Competing interests

The author(s) declare that they have no competing interests.

## Authors' contributions

KT was responsible for and involved in all parts of the work with the manuscript. KT was alone responsible for the data collection and the contacts with the nursing staff and the municipality. She is a PhD student at Lund University. Together with BN, BVS and PN she has performed the statistical analysis, instrument development and the interpretation of results. PN has particularly been involved in the statistical supervision. BN has participated in the elaboration and writing of the manuscript as an assistant supervisor. As the main supervisor BVS was responsible for the design of the study and has participated in all parts of the work with the manuscript. All authors critically reviewed and approved the final manuscript.

## Pre-publication history

The pre-publication history for this paper can be accessed here:


